# A análise da gasometria arterial prevê uma evolução desfavorável em pacientes internados com fraturas diafisárias isoladas do fêmur?

**DOI:** 10.1055/s-0045-1814128

**Published:** 2025-12-22

**Authors:** D. Sidharth, Thatchinamoorthy Santhamoorthy, Anish Anto Xavier, Lalithambigai Chellamuthu, S. Ahilan

**Affiliations:** 1 Departmento de Ortopedia, Shri Sathya Sai Medical College and Research Institute, Tamilnadu, Índia; 2 Departmento de Ortopedia, Indira Gandhi Government General Hospital and Postgraduate Institute, Puducherry, Índia; 3 Departmento de Medicina Comunitária, Mahatma Gandhi Medical College and Research Institute, Puducherry, Índia; 4 Departmento de Ortopedia, Sri Laksminarayana Institute of Medical Sciences, Puducherry, Índia

**Keywords:** admissão do paciente, análise de gases sanguíneos, fraturas do colo femoral, unidades de terapia intensiva, arterial blood gas analysis, femoral neck fractures, intensive care units, patient admission

## Abstract

**Objetivo:**

Avaliar o papel dos parâmetros da gasometria arterial (GA) na predição de desfechos desfavoráveis intra-hospitalares em pacientes com fraturas diafisárias isoladas do fêmur.

**Métodos:**

Foi realizado um estudo retrospectivo em um hospital terciário entre abril de 2020 e junho de 2021. Foram incluídos pacientes de 18 a 70 anos, de ambos os sexos, com fraturas diafisárias isoladas do fêmur. Os parâmetros da GA foram avaliados à admissão e 24 horas após a admissão. Sua associação com complicações sistêmicas, sua admissão em unidade de terapia intensiva e o tempo de internação foram avaliados.

**Resultados:**

Foram incluídos 55 pacientes, com idade média de 53,8 anos. Os parâmetros da GA à admissão e em 24 horas foram significativamente associados a desfechos desfavoráveis, especificamente pH < 7,35, bicarbonato (HCO _3_^−^ ) < 22 mmol/L, pressão parcial de dióxido de carbono (PCO _2_ ) < 35 mmHg, pressão parcial de oxigênio (PO _2_ ) < 83 mmHg e lactato > 1,8 mmol/L. Níveis elevados de lactato à admissão foram correlacionados, adicionalmente, com internação prolongada e hipertensão arterial sistêmica.

**Conclusão:**

Os parâmetros da GA avaliados à admissão e 24 horas após a admissão servem como preditores precoces de desfechos desfavoráveis em pacientes com fraturas diafisárias isoladas do fêmur. Esses achados sugerem que a GA pode ser uma ferramenta valiosa para orientar o manejo clínico desses pacientes.

## Abstract (en)

**Objective:**

To evaluate the role of arterial blood gas (ABG) parameters in predicting adverse in-hospital outcomes among patients with isolated diaphyseal femur fractures.

**Methods:**

A retrospective study was conducted at a tertiary care hospital between April 2020 and June 2021. Patients aged 18 to 70 years of both sexes with isolated diaphyseal femur fractures were included. The ABG parameters were assessed upon admission and 24 hours postadmission. Their association with systemic complications, intensive care unit admission, and length of hospital stay was evaluated.

**Results:**

The study included 55 patients with a mean age of 53.8 years. The ABG parameters upon admission and at 24 hours were significantly associated with adverse outcomes, specifically pH < 7.35, bicarbonate (HCO _3_^−^ ) < 22 mmol/L, partial pressure of carbon dioxide (PCO _2_ ) < 35 mmHg, partial pressure of oxygen (PO _2_ ) < 83 mmHg, and lactate > 1.8 mmol/L. Elevated lactate levels upon admission were additionally correlated with prolonged hospital stay and systemic arterial hypertension.

**Conclusion:**

The ABG parameters assessed upon admission and 24 hours postadmission serve as early predictors of adverse outcomes in patients with isolated diaphyseal femur fractures. These findings suggest that ABG analysis may be a valuable tool in guiding the clinical management of such patients.

## Introdução

O trauma é a principal causa de morte no mundo. 1 As mortes prematuras e a incapacidade decorrente do trauma impõem uma carga econômica substancial. 2 3 Os locais mais frequentes de trauma incluem as extremidades (particularmente fraturas ósseas longas), seguidos por lesões craniocerebrais, abdominopélvicas, torácicas e da coluna vertebral. 4 5 As fraturas de ossos longos podem ser fatais devido à hemorragia grave. 6 O conceito da “hora de ouro”, que constitui o alicerce do suporte avançado de vida ao trauma ( *advanced trauma life support* , ATLS, em inglês), enfatiza o reconhecimento precoce do choque hemorrágico e a intervenção imediata para prevenir a hipoperfusão tecidual. 7

As fraturas diafisárias do fêmur (FDFs) estão associadas a uma taxa de mortalidade aguda de 0,04%, mas podem levar a mortalidade e morbidade significativas se não forem tratadas prontamente nas horas iniciais. 8 As complicações sistêmicas comumente associadas a essas fraturas incluem dessaturação transitória (DT), choque hemorrágico, síndrome da embolia gordurosa (SEG), síndrome do desconforto respiratório agudo (SDRA) e tromboembolismo pulmonar (TEP). Portanto, a identificação precoce de indicadores prognósticos é fundamental para a prevenção dessas complicações.

Atualmente, contamos com os sinais vitais tradicionais, como pressão arterial e frequência cardíaca, para orientar o exame inicial dessas fraturas. No entanto, os sinais vitais devem ser apenas marcadores substitutos, e não medidas diretas de fornecimento de oxigênio. Melhores marcadores da presença e da extensão da hemorragia em curso podem ser os produtos do metabolismo resultantes da hipoperfusão tecidual, como o déficit de base (DB) e o nível sérico de lactato (NL). 9 10 Estudos mostraram que a mortalidade de pacientes com trauma pode ser prevista com base nos níveis de déficit de base nas primeiras 24 horas. 11 12 13

Os parâmetros da gasometria arterial (GA) foram identificados como preditores úteis da gravidade da lesão cerebral traumática e para a identificação dos desfechos do paciente. 14 15 No trauma ortopédico, apenas alguns estudos examinaram o papel prognóstico dos parâmetros da GA, e esses foram conduzidos principalmente em pacientes com fraturas pélvicas isoladas ou associadas a fraturas de ossos longos. 16 17 Até onde sabemos, nenhum estudo avaliou especificamente o papel dos parâmetros da GA na previsão de desfechos hospitalares desfavoráveis em pacientes com FDF isolada.

Portanto, o objetivo primário deste estudo foi avaliar o papel de diversos parâmetros da GA na previsão de desfechos desfavoráveis intra-hospitalares em pacientes com FDFs isoladas. O objetivo secundário foi analisar a associação de fatores demográficos, comorbidades e parâmetros da GA na população estudada.

## Materiais e Métodos

Este estudo observacional retrospectivo foi realizado no Departamento de Ortopedia de um hospital terciário entre abril de 2020 e junho de 2021.

Os critérios de inclusão foram pacientes com idade entre 18 e 70 anos, com FDF isolada, aberta ou fechada, ou fratura subtrocantérica do fêmur (FSF, unilateral ou bilateral), ocorrida dentro de 48 horas do trauma.

Foram excluídos casos com apresentação após 48 horas, traumatismo craniano grave (pontuação na Escala de Coma de Glasgow < 8), sangramento intracraniano, politrauma, lesões torácicas, parada cardíaca pré-hospitalar, estabilização prévia/oxigenoterapia em outro local e condições preexistentes que afetam os NLs (insuficiência cardíaca, doença pulmonar obstrutiva crônica, doença hepática, infecção por doença do coronavírus 2019 ( *coronavirus disease 2019* , COVID-19, em inglês) ativa ou terapia antirretroviral).

Os dados coletados retrospectivamente incluíram idade, sexo, mecanismo do trauma, tipo de fratura, tempo desde a lesão, comorbidades, admissão na Unidade de Terapia Intensiva (UTI), necessidade de suporte ventilatório à admissão, tempo de internação e complicações sistêmicas. Complicações sistêmicas foram definidas como a ocorrência de sepse, lesão renal aguda, SDRA, síndrome de disfunção de múltiplos órgãos (SDMO), trombose venosa profunda (TVP), TEP ou infarto do miocárdio durante a hospitalização.

A análise da GA foi realizada em dois tempos padronizados: à admissão (dentro de 2 horas após a chegada ao hospital) e 24 horas após a admissão usando o sistema Cobas b 221 (Roche Diagnostics). Os parâmetros registrados incluíram pH, bicarbonato (HCO _3_^−^ ), pressão parcial de dióxido de carbono (PCO _2_ ), pressão parcial de oxigênio (PO _2_ ), NL e excesso de base.

As faixas de referência foram pH: 7,35 a 7,45; HCO _3_^−^ : 22 a 26 mEq/L; PCO _2_ : 35 a 45 mmHg; PO _2_ : 75 a 100 mmHg; NL: 0,20 a 1,80 mmol/L; e excesso de base: −2 a +2 mmol/L. Valores anormais foram definidos como: pH < 7,35 (acidemia) ou > 7,45 (alcalemia); PCO _2_ > 45 mmHg com pH < 7,35 (acidose respiratória), ou PCO _2_ < 35 mmHg com pH > 7,45 (alcalose respiratória); HCO _3_^−^ < 22 mEq/L com pH < 7,35 (acidose metabólica), ou HCO _3_^−^ > 26 mEq/L com pH >7,45 (alcalose metabólica); excesso de base < −2 ou > +2 mmol/L; e NL > 1,80 mmol/L (acidose lática).

Os dados foram inseridos no software Epicollect 5 (Centre for Genomic Pathogen Surveillance) e analisados no IBM SPSS Statistics for Windows (IBM Corp.), versão 26. A estatística descritiva foi apresentada como média ± desvio padrão (DP) para as variáveis contínuas e como frequências com percentuais para as variáveis categóricas. A normalidade das variáveis contínuas foi avaliada pelos testes de Kolmogorov–Smirnov e Shapiro–Wilk, com *p* > 0,05 considerada distribuição normal, corroborada por gráficos quantil-quantil (Q–Q). As associações entre os parâmetros da GA e as variáveis demográficas ou clínicas foram avaliadas pelo teste do Qui-quadrado. A regressão logística multivariada foi realizada para identificar preditores independentes de complicações sistêmicas, considerando anormalidades da GA, o mecanismo do trauma e as comorbidades do paciente. Valores de *p* < 0,05 foram considerados estatisticamente significativos.

O cálculo do tamanho da amostra baseou-se em uma incidência presumida de 25% de complicações sistêmicas entre pacientes com fratura de fêmur. Para detectar uma diferença significativa com poder de 80% e nível de confiança de 95%, foi necessário um mínimo de 120 registros de pacientes. A aprovação do Comitê de Ética Institucional foi obtida antes do início do estudo, com o número de referência: CSP-MED/21/NOV/72/138. Como este foi um estudo retrospectivo, com dados anônimos de registros existentes, a exigência de consentimento informado foi dispensada pela instituição.

## Resultados

Dentre a população estudada, 29 pacientes (52,7%) apresentaram fratura subtrocantérica e 26 pacientes (47,3%) apresentaram fratura da diáfise. O sexo masculino predominou (n = 31, 56,4%) em relação ao feminino (n = 24, 43,6%). A média de idade foi de 53,84 ± 17,75 anos, sendo a maior proporção de pacientes (n = 21, 38,2%) na faixa etária de 66 a 70 anos, seguida por 16 (29,1%) na faixa etária de 51 a 65 anos, 10 (18,2%) na faixa etária de 31 a 50 anos e 8 (14,5%) na faixa etária de 18 a 30 anos.

Escorregamento e queda foi o mecanismo de lesão mais comum (n = 30, 54,5%), seguido por acidentes de trânsito (n = 25, 45,5%). As fraturas do fêmur direito (n = 36, 65,5%) foram mais comuns do que as do lado esquerdo (n = 19, 34,5%). O atraso na análise da GA, a partir do momento da lesão, foi distribuído da seguinte forma: 1 a 3 horas em 17 pacientes (30,9%), 4 a 6 horas em 16 (29,1%), 7 a 9 horas em 11 (20,0%), 10 a 12 horas em 6 (10,9%) e mais de 12 horas em 5 (9,1%). Não foram observadas associações significativas entre os parâmetros da GA e as variáveis demográficas (idade e sexo). Da mesma forma, não foram identificadas associações significativas entre os parâmetros da GA e fatores clínicos (tipo de fratura, mecanismo do trauma, lado da lesão ou tempo desde a lesão).

Da população do estudo, 21 pacientes (38,2%) não apresentavam comorbidades, 18 (32,7%) apresentavam hipertensão arterial sistêmica (HAS) isolada e 16 (29,1%) apresentavam múltiplas comorbidades, como diabetes mellitus (DM), HAS e hipotireoidismo. Foram comparados os parâmetros da GA entre pacientes com e sem essas comorbidades. Dentre os parâmetros analisados, observaram-se diferenças significativas nos NLs entre hipertensos e não hipertensos ( Tabela 1 ). Na admissão, os pacientes com HAS apresentaram NLs significativamente mais elevados (> 1,8 mmol/L) em comparação com os pacientes não hipertensos ( *p* = 0,013). Essa diferença persistiu por 24 horas, com os NLs mantidos significativamente elevados no grupo de hipertensos ( *p* < 0,0001). Nenhum outro parâmetro da GA apresentou variação significativa entre os grupos de comorbidades (DM, HAS e hipotireoidismo).

**Tabela 1:** Associação entre os parâmetros da GA e HAS

Variáveis	HAS	Sem HAS	Valor de *p* *
**pH à admissão**			
< 7,35	5	9	0,402
7,35–7,45	18	20	
> 7,45	1	2	
**pH em 24 horas**			
< 7,35	3	9	0,107
7,35–7,45	19	21	
> 7,45	2	1	
** HCO _3_^−^ à admissão (mEq/L) **			
< 22	10	14	0,082
22–26	10	16	
> 26	4	1	
** HCO _3_^−^ em 24 horas (mEq/L) **			
< 22	9	10	0,053
22–26	14	14	
> 26	1	7	
** PCO _2_ à admissão (mmHg) **			
< 35	4	5	0,155
35–45	19	21	
> 45	1	5	
** PCO _2_ em 24 horas (mmHg) **			
< 35	4	7	0,052
35–45	19	11	
> 45	1	3	
** PO ^2^ à admissão (mmHg) **			
< 83	11	20	0,068
83–108	9	5	
> 108	4	6	
** PO _2_ em 24 horas (mmHg) **			
< 83	4	10	0,154
83–108	17	19	
> 108	3	2	
**Base à admissão (mmol/L)**			
Déficit de base (< −2,0)	11	12	0,143
Normal (−2.0– + 2,0)	12	18	
Excesso de base (> 2,0)	1	1	
**Base em 24 horas (mmol/L)**			
Déficit de base (< −2,0)	10	12	0,751
Normal (−2.0– + 2,0)	13	18	
Excesso de base (> 2,0)	1	1	
**NL à admissão (mmol/L)**			
0,2–1,8	4	26	0,013**
> 1,8	20	5	
**NL em 24 horas (mmol/L)**			
0,2–1,8	6	19	< 0,001**
> 1,8	18	2	

**Abreviações:** GA, gasometria arterial; HCO _3_^−^ , bicarbonato; HAS, hipertensão arterial sistêmica; NL, nível sérico de lactato; PCO _2_ , pressão parcial de dióxido de carbono; PO _2_ , pressão parcial de oxigênio. **Notas:** *Teste do Qui-quadrado; **estatisticamente significativo ( *p* < 0,05).

Dentre os 55 pacientes do estudo, 19 (34,5%) necessitaram de internação na UTI, dos quais 9 (47,4%) apresentaram FDF e 10 (52,6%) apresentaram FSF. Não foi observada associação significativa entre a admissão na UTI e o tipo de fratura.

A DT foi a complicação mais comum, ocorrendo em 19 pacientes (34,5%) nos grupos de fraturas de diáfise e subtrocantéricas. Seguiu-se a síndrome da embolia gordurosa (SEG) e a SDRA, cada uma relatada em 4 pacientes (7,3%). O tromboembolismo pulmonar (TEP) foi observado em apenas 1 paciente (1,8%) com fratura subtrocantérica. Não foi encontrada associação significativa entre as complicações sistêmicas e o tipo de fratura.

Entre os pacientes com FDF, 14 (53,8%) tiveram internação de < 10 dias, enquanto 12 (46,2%) permaneceram ≥ 10 dias. Entre os pacientes com FSF, 17 (58,6%) tiveram permanência < 10 dias e 12 (41,4%) ≥ 10 dias. No entanto, não foi observada associação estatisticamente significativa entre o tipo de fratura e o tempo de internação.

As Tabelas 2 , 3 e 4 apresentam as associações entre os parâmetros da GA e as complicações sistêmicas, a admissão na UTI e o tempo de internação. Pacientes com complicações sistêmicas demonstraram anormalidades significativas em vários parâmetros da GA: 1) níveis mais baixos de pH em 24 horas ( *p* = 0,026), indicando acidose contínua; 2) níveis reduzidos de HCO _3_^−^ tanto à admissão ( *p* = 0,019) quanto em 24 horas ( *p* = 0,025), sugerindo compensação metabólica inadequada; 3) NLs elevados à admissão ( *p* = 0,038); 4) oxigenação prejudicada com menor PO _2_ à admissão ( *p* = 0,048) e em 24 horas ( *p* < 0,0001); e 5) diferenças significativas nos níveis de PCO _2_ à admissão ( *p* = 0,011) e em 24 horas ( *p* = 0,029), refletindo distúrbios respiratórios e metabólicos combinados.

**Tabela 2:** Associação entre os parâmetros da GA e complicações sistêmicas

Variáveis	Sem complicações sistêmicas	Complicações sistêmicas	Valor de *p* *
**pH à admissão**			
< 7,35	4	10	0,146
7,35–7,45	19	19	
> 7,45	1	2	
**pH em 24 horas**			
< 7,35	2	10	0,0260**
7,35–7,45	25	16	
> 7,45	1	1	
** HCO _3_^−^ à admissão (mEq/L) **			
< 22	8	16	0,019**
22–26	18	8	
> 26	4	1	
** HCO _3_^−^ em 24 horas (mEq/L) **			
< 22	6	13	0,025**
22–26	20	8	
> 26	4	4	
** PCO _2_ à admissão (mmHg) **			
<35	1	8	0,011**
35–45	26	14	
> 45	4	2	
** PCO _2_ em 24 horas (mmHg) **			
< 35	2	9	0,029**
35–45	24	16	
> 45	1	3	
** PO _2_ à admissão (mmHg) **			
< 83	15	16	0,048**
83–108	12	2	
> 108	2	8	
** PO _2_ em 24 horas (mmHg) **			
< 83	1	13	0,001**
83–108	26	10	
> 108	1	4	
**Base à admissão (mmol/L)**			
Déficit de base (< −2,0)	9	14	0,388
Normal (−2.0– + 2,0)	17	13	
Excesso de base (> 2,0)	1	1	
**Base em 24 horas (mmol/L)**			
Deficit de base (< −2,0)	7	15	0,168
Normal (−2.0– + 2,0)	18	13	
Excesso de base (> 2,0)	1	1	
**NL à admissão (mmol/L)**			
0,2–1,8	18	12	0,038**
> 1,8	8	17	
**NL em 24 horas (mmol/L)**			
0,2–1,8	20	14	0,417
> 1,8	10	11	

**Abreviações:** GA, gasometria arterial; HCO _3_^−^ , bicarbonato; NL, nível sérico de lactato; PCO _2_ , pressão parcial de dióxido de carbono; PO _2_ , pressão parcial de oxigênio. **Notas:** *Teste do Qui-quadrado; **estatisticamente significativo ( *p* < 0,05).

**Tabela 3:** Associação entre os parâmetros da GA e admissão na UTI

Variáveis	Não-UTI	UTI	Valor de *p* *
**pH à admissão**			
< 7,35	6	8	0,038**
7,35–7,45	29	9	
> 7,45	1	2	
**pH em 24 horas**			
< 7,35	6	8	0,006**
7,35–7,45	17	8	
> 7,45	1	1	
** HCO _3_^−^ à admissão (mEq/L) **			
< 22	14	10	0,636
22–26	17	9	
> 26	4	1	
** HCO _3_^−^ em 24 horas (mEq/L) **			
< 22	7	12	0,004**
22–26	23	5	
> 26	6	2	
** PCO _2_ à admissão (mmHg) **			
<35	3	6	0,052
35–45	29	11	
> 45	5	1	
** PCO _2_ em 24 horas (mmHg) **			
< 35	4	7	0,046**
35–45	30	10	
> 45	2	2	
** PO _2_ à admissão (mmHg) **			
< 83	18	13	0,039**
83–108	13	1	
> 108	5	5	
** PO _2_ em 24 horas (mmHg) **			
< 83	3	11	0,001**
83–108	32	4	
> 108	1	4	
**Base à admissão (mmol/L)**			
Déficit de base (< −2,0)	13	10	0,261
Normal (−2.0– + 2,0)	23	7	
Excesso de base (> 2,0)	1	1	
**Base em 24 horas (mmol/L)**			
Déficit de base (< −2,0)	10	12	0,053
Normal (−2.0– + 2,0)	24	7	
Excesso de base (> 2,0)	1	1	
**NL à admissão (mmol/L)**			
0,2–1,8	23	7	0,027**
> 1,8	12	13	
**NL em 24 horas (mmol/L)**			
0.2–1.8	24	10	0,308
> 1.8	12	9	

**Abreviações:** GA, gasometria arterial; HCO _3_^−^ , bicarbonato; NL, nível sérico de lactato; PCO _2_ , pressão parcial de dióxido de carbono; PO _2_ , pressão parcial de oxigênio. **Notas:** *Teste do Qui-quadrado; **estatisticamente significativo ( *p* < 0,05).

**Tabela 4:** Associação entre os parâmetros da GA e internação

Variáveis	< 10 dias de internação	≥ 10 dias de internação	Valor de *p* *
**pH à admissão**			
< 7,35	6	8	0,038**
7,35–7,45	29	9	
> 7,45	1	2	
**pH em 24 horas**			
< 7,35	6	8	0,006**
7,35–7,45	17	8	
> 7,45	1	1	
** HCO _3_^−^ à admissão (mEq/L) **			
< 22	14	10	0,636
22–26	17	9	
> 26	4	1	
** HCO _3_^−^ em 24 horas (mEq/L) **			
< 22	7	12	0,004**
22–26	23	5	
> 26	6	2	
** PCO _2_ à admissão (mmHg) **			
< 35	3	6	0,052
35–45	29	11	
> 45	5	1	
** PCO _2_ em 24 horas (mmHg) **			
< 35	4	7	0,046**
35–45	30	10	
> 45	2	2	
** PO _2_ à admissão (mmHg) **			
< 83	18	13	0,039**
83–108	13	1	
> 108	5	5	
** PO _2_ em 24 horas (mmHg) **			
< 83	3	11	0,001**
83–108	32	4	
> 108	1	4	
**Base à admissão (mmol/L)**			
Déficit de base (< −2,0)	13	10	0,261
Normal (−2.0– + 2,0)	23	7	
Excesso de base (> 2,0)	1	1	
**Base em 24 horas (mmol/L)**			
Déficit de base (< −2,0)	10	12	0,053
Normal (−2.0– + 2,0)	24	7	
Excesso de base (> 2,0)	1	1	
**NL à admissão (mmol/L)**			
0,2–1,8	23	7	0,027**
> 1,8	12	13	
**NL em 24 horas (mmol/L)**			
0,2–1,8	24	10	0,308
> 1,8	12	9	

**Abreviações:** GA, gasometria arterial; HCO _3_^−^ , bicarbonato; NL, nível sérico de lactato; PCO _2_ , pressão parcial de dióxido de carbono; PO _2_ , pressão parcial de oxigênio. **Notas:** *Teste do Qui-quadrado; **estatisticamente significativo ( *p* < 0,05).

Os pacientes que necessitaram de admissão na UTI apresentaram alterações significativos nos parâmetros da AG: 1) níveis mais baixos de pH à admissão ( *p* = 0,038) e 24 horas ( *p* = 0,006), refletindo acidose persistente; 2) níveis reduzidos de HCO _3_^−^ em 24 horas ( *p* = 0,004), indicando compensação metabólica inadequada; 3) NLs elevados à admissão ( *p* = 0,027); 4) oxigenação prejudicada com menor PO _2_ à admissão ( *p* = 0,039) e em 24 horas ( *p* < 0,0001); e 5) diferenças significativas na PCO _2_ em 24 horas ( *p* = 0,046), sugerindo distúrbios respiratórios e metabólicos combinados associados à necessidade de UTI.

O tempo de internação prolongado foi associado a anormalidades significativas da AG: 1) níveis mais baixos de pH à admissão ( *p* = 0,003) e 24 horas ( *p* = 0,001), indicando acidose persistente; 2) NLs elevados à admissão ( *p* = 0,005); e 3) oxigenação prejudicada (PO _2_ ) em 24 horas ( *p* = 0,038), refletindo comprometimento metabólico e respiratório contínuo. Embora HCO _3_^−^ e PCO _2_ não tenham atingido significância estatística, apresentaram tendência a valores anormais em pacientes com internação prolongada.

## Discussão

As fraturas pélvicas e de ossos longos, particularmente as da diáfise femoral, são lesões graves devido à sua proximidade com as principais estruturas vasculares e ao efeito de fortes contrações musculares, que exacerbam o dano tecidual. 18 A hemorragia dessas fraturas pode levar rapidamente ao choque, enquanto complicações como SEG, SDRA, pneumonia e TEP contribuem significativamente para a morbidade e mortalidade. Pacientes com fraturas de ossos longos foram relatados como tendo risco 2,35 vezes maior de SDRA no período inicial do hospital. As anormalidades da GA foram identificadas como indicadores confiáveis da gravidade do trauma. 19 20 21 22 23 Este estudo teve como objetivo avaliar a relevância clínica desses parâmetros na previsão de desfechos desfavoráveis em pacientes com FDFs isoladas.

A falta de correlação entre os valores da GA e as variáveis demográficas ou relacionadas à fratura neste estudo indica que os desfechos desfavoráveis não são determinados apenas por idade, sexo ou tipo de fratura. Em vez disso, as respostas ao estresse sistêmico e metabólico, refletidas em alterações da GA, parecem desempenhar um papel central na determinação da progressão clínica.

Uma observação notável foi a forte associação entre HAS e NL elevado à admissão e em 24 horas. A hipertensão é conhecida por causar disfunção endotelial e comprometimento microvascular, o que pode reduzir a perfusão tecidual e retardar a depuração de NL no trauma agudo. Esse achado é consistente com estudos anteriores que demonstraram o impacto prognóstico do NL e dos marcadores de perfusão em pacientes com trauma. 24 25

Mohsenian et al. 17 destacaram o valor prognóstico de índices da GA, como a saturação de O _2_ , o excesso de base e o PCO _2_ em pacientes com fraturas femorais e pélvicas. Concordando com suas observações, o presente estudo ressalta que anormalidades em PO _2_ , pH e NL podem servir como sinais de alerta precoce para complicações sistêmicas e necessidades de cuidados críticos. Embora a mortalidade não tenha sido observada nesta coorte, um declínio na oxigenação e na compensação metabólica refletiu as tendências descritas em estudos anteriores, sugerindo que o monitoramento da GA pode fornecer informações prognósticas mesmo em casos isolados de fratura.

Curiosamente, hipocapnia, em vez de hipercapnia, foi observada em associação com desfechos desfavoráveis. Isso provavelmente reflete a hiperventilação compensatória em resposta à acidose metabólica. Clinicamente, esse achado é importante porque a hipocapnia pode mascarar a gravidade do estresse metabólico quando interpretada isoladamente. Reconhecer esse mecanismo compensatório é essencial, pois permite identificar pacientes com alto risco de deterioração, apesar de parâmetros de ventilação aparentemente normais. 26 Ross et al. 27 demonstraram anteriormente que a acidose grave (pH < 7,0) se correlaciona com desfechos ruins, e nossos achados reforçam o conceito de que alterações ainda mais leves no pH (< 7,35) não devem ser negligenciadas no atendimento ao trauma.

Estudos anteriores enfatizaram o valor prognóstico do NL e de déficit de base em pacientes com trauma. Ward et al. 28 relataram sobrevida significativamente menor entre pacientes com NL elevado ou déficit de base inicial, enquanto Oladipo et al. 29 associaram essas elevações iniciais à morbidade pós-operatória e hospitalização prolongada em trauma ortopédico. Essas observações se alinham aos achados desse estudo, em que o NL elevado e o equilíbrio ácido–base comprometido foram preditivos de desfechos desfavoráveis e de permanência hospitalar prolongada.

A alta incidência de hipoxemia inaparente, particularmente DT, destaca ainda mais as complicações respiratórias subclínicas que acompanham as fraturas de ossos longos. Rosenkrantz et al. 30 documentaram padrões semelhantes, atribuindo-os à SEG, à obstrução microvascular e às respostas inflamatórias. Isso ressalta a importância do monitoramento rotineiro de oxigênio e de avaliações seriadas da GA em pacientes com fratura aparentemente estável.

Esses achados, em conjunto, sugerem que a análise da GA não é apenas um adjuvante laboratorial, mas também uma ferramenta clinicamente relevante para a estratificação precoce do risco em fraturas femorais. Alterações persistentes no pH, NL e PO _2_ refletem oxigenação tecidual inadequada e mecanismos compensatórios comprometidos, o que pode orientar decisões sobre monitoramento de UTI, ressuscitação agressiva e vigilância de complicações.

O presente estudo apresenta algumas limitações. Seu caráter retrospectivo e o tamanho da amostra podem limitar a generalização. Não foram avaliados o impacto do momento da fixação cirúrgica nem o papel do alargamento intramedular como contribuintes das alterações da GA. Estudos prospectivos que incorporam monitoramento da GA perioperatório em série podem esclarecer ainda mais esses aspectos.

## Conclusão

Verificou-se que os valores da GA à admissão e 24 horas após a admissão desempenham um papel na previsão precoce de desfechos desfavoráveis em pacientes com FDF isoladas. Portanto, o monitoramento rotineiro dos parâmetros da GA é recomendado nesses pacientes. Dada a natureza retrospectiva e o tamanho limitado da amostra deste estudo, estudos prospectivos com coortes maiores são necessários para validar esses achados.
